# Role of lactic acid bacteria and yeasts in sourdough fermentation during breadmaking: Evaluation of postbiotic-like components and health benefits

**DOI:** 10.3389/fmicb.2022.969460

**Published:** 2022-09-08

**Authors:** Omar Pérez-Alvarado, Andrea Zepeda-Hernández, Luis Eduardo Garcia-Amezquita, Teresa Requena, Gabriel Vinderola, Tomás García-Cayuela

**Affiliations:** ^1^Tecnologico de Monterrey, Escuela de Ingeniería y Ciencias, Food and Biotech Lab, Zapopan, Jalisco, Mexico; ^2^Department of Food Biotechnology and Microbiology, Institute of Food Science Research, CIAL (CSIC), Madrid, Spain; ^3^Faculty of Chemical Engineering, Instituto de Lactología Industrial (CONICET-UNL), National University of Litoral, Santa Fe, Argentina

**Keywords:** sourdough, postbiotics, functional bread, backslopping, baking

## Abstract

Sourdough (SD) fermentation is a traditional biotechnological process used to improve the properties of baked goods. Nowadays, SD fermentation is studied for its potential health effects due to the presence of postbiotic-like components, which refer to a group of inanimate microorganisms and/or their components that confer health benefits on the host. Some postbiotic-like components reported in SD are non-viable microorganisms, short-chain fatty acids, bacteriocins, biosurfactants, secreted proteins/peptides, amino acids, flavonoids, exopolysaccharides, and other molecules. Temperature, pH, fermentation time, and the composition of lactic acid bacteria and yeasts in SD can impact the nutritional and sensory properties of bread and the postbiotic-like effect. Many *in vivo* studies in humans have associated the consumption of SD bread with higher satiety, lower glycemic responses, increased postprandial concentrations of short-chain fatty acids, and improvement in the symptoms of metabolic or gastrointestinal-related diseases. This review highlights the role of bacteria and yeasts used for SD, the formation of postbiotic-like components affected by SD fermentation and the baking process, and the implications of functional SD bread intake for human health. There are few studies characterizing the stability and properties of postbiotic-like components after the baking process. Therefore, further research is necessary to develop SD bread with postbiotic-related health benefits.

## Introduction

Sourdough (SD) fermentation is a well-known biotechnological process that has been in use for 5,000 years and has shown the ability to improve the sensory, rheological, and shelf-life properties of baked goods. This biotechnology process encompasses a great variety of lactic acid bacteria (LAB) and yeast interactions (Poutanen et al., 2009). Sourdough is the result of fermenting a mixture of flour, water, and other ingredients by LAB and yeasts naturally occurring in the flour. Sourdough is propagated during backslopping process, in which a new mixture of flour and water is fermented by using as a starter the SD from a previous fermentation batch (Rizzello et al., 2019). From a microbiological point of view, SD is an ecosystem characterized by an environment with a low pH, high carbohydrate concentration, oxygen limitation, and a LAB cell count exceeding that of yeasts (De Vuyst et al., 2014). Lactic acid bacteria dominate the mature SD, while the yeast content is one/two logarithmic cycles lower (Rizzello et al., 2019). The major metabolic activities of the SD microbiota are acidification (LAB), flavor formation (LAB and yeasts), and leavening (yeasts and heterofermentative LAB species; De Vuyst et al., 2014). Even though LAB and yeasts originate principally from the flour and environmental microbiota, the process of microbiota maturation during SD fermentation depends on various factors such as temperature, the chemical and enzymatic composition of the flour, redox potential, water content, and time (Rizzello et al., 2019).

Nowadays, the culture-based techniques used to characterize SD microbial diversity across studies investigating the distribution of SD bacterial and fungal taxa are variable and biased (De Vuyst et al., 2014; Michel et al., 2019; Landis et al., 2021). Sourdough starters are maintained in many households, but these differ from those in bakeries due to heterogeneity among environments, production practices, and ingredients (Landis et al., 2021). The types/origins of the flours and baking procedures seem to affect mainly the SD biodiversity. Different SDs from the same region may be similar in composition due to the response to regional microclimates or the restricted dispersion of microbes (Landis et al., 2021). Bread producers often attribute distinct regional properties to their breads, giving credit to the environment for their unique characteristics (Landis et al., 2021). Some of the microorganisms identified from SD microbiota characterization studies are shown in Table 1, differentiated by LAB (homofermentative, obligate heterofermentative, and facultative heterofermentative) and yeasts (De Vuyst and Neysens, 2005; Chavan and Chavan, 2011; Gänzle and Gobbetti, 2013; De Vuyst et al., 2017; Papadimitriou et al., 2019; Preedy and Watson, 2019; Arora et al., 2021).

**Table 1:** Lactic acid bacteria (LAB) and yeasts found in different types of sourdough.

**Homofermentative LAB**	**Obligate heterofermentative LAB**	**Facultative heterofermentative LAB**	**Yeasts**
*Enterococcus casseliflavus* *Enterococcus durans* *Enterococcus faecalis* *Enterococcus faecium* *Lactobacillus amylovorus* *Lactobacillus amylolyticus* *Lactobacillus delbrueckii* *Lactobacillus acidophilus* *Companilactobacillus farciminis* *Lactobacillus johnsonii* *Companilactobacillus crustorum Companilactobacillus heilongjiangensis Companilactobacillus mindensis Companilactobacillus nantensis Companilactobacillus nodensis* *Lactobacillus crispatus* *Lactobacillus gallinarum* *Lactobacillus gasseri* *Lactobacillus helveticus* *Liquorilactobacillus nagelii* *Ligilactobacillus salivarius, Streptococcus constellatus* *Streptococcus equinus* *Streptococcus suis*	*Levilactobacillus acidifarinae, Levilactobacillus brevis, Limosilactobacillus fermentum Limosilactobacillus reuteri, Limosilactobacillus pontis, Furfurilactobacillus rossiae Limosilactobacillus panis, Companilactobacillus crustorum* *Latilactobacillus curvatus* *Limosilactobacillus frumenti, Fructilactobacillus fructivorans* *Levilactobacillus hammesii* *Levilactobacillus koreensis, Levilactobacillus namurensis* *Companilactobacillus* *nodensis* *Limosilactobacillus oris, Lentilactobacillus parabuchneri* *Fructilactobacillus sanfranciscensis* *Limosilactobacillus secaliphilus* *Furfurilactobacillus siliginis* *Lentilactobacillus buchneri, Fructilactobacillus fructivorans* *Lentilactobacillus hilgardii* *Fructilactobacillus fructivorans* *Lentilactobacillus kefiri, Apilactobacillus kunkeei, Fructilactobacillus lindneri Limosilactobacillus mucosae, Limosilactobacillus fermentum, Secundilactobacillus collinoides Limosilactobacillus vaginalis, Levilactobacillus zymae, Leuconostoc citreum* *Leuconostoc gelidum* *Leuconostoc mesenteroides* *Weissella cibaria* *Weissella confusa* *Weissella hellenica* *Weissella kandleri*	*Companilactobacillus alimentarius* *Companilactobacillus paralimentarius Lactiplantibacillus plantarum* *Lacticaseibacillus casei, Lacticaseibacillus paracasei Lacticaseibacillus rhamnosus* *Levilactobacillus spicheri Lactiplantibacillus xiangfangensis* *Limosilactobacillus coleohominis* *Companilactobacillus kimchii* *Lactiplantibacillus pentosus Schleiferilactobacillus perolens* *Latilactobacillus sakei* *Pediococcus acidilactici* *Lapidilactobacillus dextrinicus* *Pediococcus pentosaceus*	*Saccharomyces cerevisiae* *Saccharomyces bayanus* *Kazachstania exigua* *Kazachstania humilis* *Kazachstania servazzi* *Kazachstania exigua* *Pichia kudriavzevii* *Torulaspora delbrueckii* *Wickerhamomyces anomalus* *Pichia kudriavzevii* *Candida tropicalis* *Candida glabrata* *Candida krusei* *Candida pelicullosa* *Yarrowia keelungensis* *Torulaspora delbrueckii* *Rhodotorula mucilaginosa*

The microorganisms in the table were compiled from the following sourdough microbiota characterization: De Vuyst and Neysens (2005), Chavan and Chavan (2011), Gänzle and Gobbetti (2013), De Vuyst et al. (2017), Papadimitriou et al. (2019), Preedy and Watson (2019), and Arora et al. (2021).

Fermented foods and functional ingredients (such as probiotics, prebiotics, and synbiotics) can be used as dietary interventions seeking health benefits. Probiotics are live microorganisms that confer a health benefit when administered in adequate amounts. Probiotic products may also deliver significant amounts of non-viable cells due to cell death during storage (Salminen et al., 2021). The interest in the potential effect of non-viable microorganisms and their components on health is rising. Fermented foods can contain numerous non-viable cells, especially after prolonged storage, thermal treatments, or processes (such as pasteurization or baking). Fermentation mediated by LAB produces different cellular structures and metabolites, such as cell surface components, lactic acid, short-chain fatty acids (SCFAs), and bioactive peptides, among other effector molecules associated with benefits to human health (Salminen et al., 2021).

New terms have been used to name these non-viable microbial cells and metabolites in recent years, including paraprobiotics, parapsychobiotics, ghost probiotics, metabiotics, tyndallized probiotics, and bacterial lysates. However, the concept of postbiotics to promote health is emerging as an important microorganism-derived tool (Salminen et al., 2021). The term postbiotic can be considered as a composite of biotic, defined as “relating to or resulting from living organisms,” and post, which refers to “after life,” implying that postbiotics are non-living microorganisms. Thus, postbiotics are defined as a preparation of inanimate microorganisms and/or their components that provides benefits to the host’s health (Salminen et al., 2021). The definition of postbiotics requires identification to the strain level of the microbe/s used for their preparation, a deliberate termination of cell viability step, and the demonstration of a health benefit in a well-designed and conducted efficacy trial in the target host (Salminen et al., 2021). Since SD fermentation is performed by microbes that occur naturally in the flours used to prepare the dough, and even though the baking step represents a deliberate termination of cell viability, the presence of a consortium of unidentified microbes prevents SD from being regarded as a postbiotic. Nevertheless, metagenomic/metataxonomic techniques based in Next Generation Sequencing are now widely used to identify the microbiota of SDs worldwide and, in this sense, the microbes would be not “completely unidentified. Moreover, in inoculated SDs some postbiotic-like substances can be correlated or assigned to specific dominant or co-dominant LAB and/or yeast strains that are imposed on the native microbiota. Based on the above mentioned, the term “postbiotic-like” will be used in this review. This term has recently employed to refer the use of cell-free supernatants of lactic acid bacteria (Spaggiari et al., 2022).

The advantage of postbiotic over probiotic microorganisms is that they present little or no interaction with the different compounds in the food matrix, which increases shelf-life and maintains the same sensory and physicochemical properties. Other advantages are that they remain stable over a wide range of pH and temperature, which allows ingredients with higher acidity to be added and treated by thermal processing in a way that their functionality is not compromised, minimizing the chances of microbial contamination after packaging and during storage (Nataraj et al., 2020). Currently, researchers are particularly focused on the discovery and characterization of new LAB strains able to biosynthesize active compounds such as exopolysaccharides (EPS), antimicrobial compounds, bioactive peptides, and SCFAs to exploit their functional properties in food (Păcularu-Burada et al., 2020b). Moreover, the selection of LAB and yeast strains (Table 1) to further design starter cultures must also consider important features, such as the preservation technologies and overall nutritional-functional aspects of the final fermented products. This review focuses on the roles of LAB and yeasts in SD in the production of baked goods with enhanced properties and the presence of postbiotic-like components. The role of LAB in the formation of postbiotic-like components during SD fermentation and baking, as well as the health benefits of SD bread, are also explored. In this respect, the timeline for the literature search was set to the last 10 and 20 years using “postbiotic” and “sourdough” as main keywords, respectively. The screening strategy included full text research articles dealing with microbiological, biochemical, technological and/or health features of traditional SD baked goods.

## Role of sourdough fermentation in the production of baked goods

Bread is one of the most important staple foods consumed in the world (Koistinen et al., 2018). Its recipe comprises cereal flour and can include pseudocereals and/or legumes, water, salt, other minor ingredients, and a leavening agent (Rizzello et al., 2019). Cereals and legumes are valuable sources of proteins, fats, and dietary fiber. Through lactic acid fermentation, the properties of these ingredients can be improved, and the sensory characteristics of the final products enhanced. In recent years, the use of SD has become increasingly standardized, and the interaction among microbial cultures has been studied with the aim of employing fermentation in baking for leavening, flavor formation, and improving stability (Gobbetti et al., 2019). Moreover, SD fermented with LAB is a source of proteolytic enzymes, activated by acid production, that are likely to eliminate gluten toxicity during bread making (Fekri et al., 2020). Furthermore, the phytic acid and other antinutritional factors from cereals and legumes are reduced by specific enzymes produced during fermentation, resulting in higher bioavailability of important minerals in baked goods (Fekri et al., 2020).

**Figure 1:**
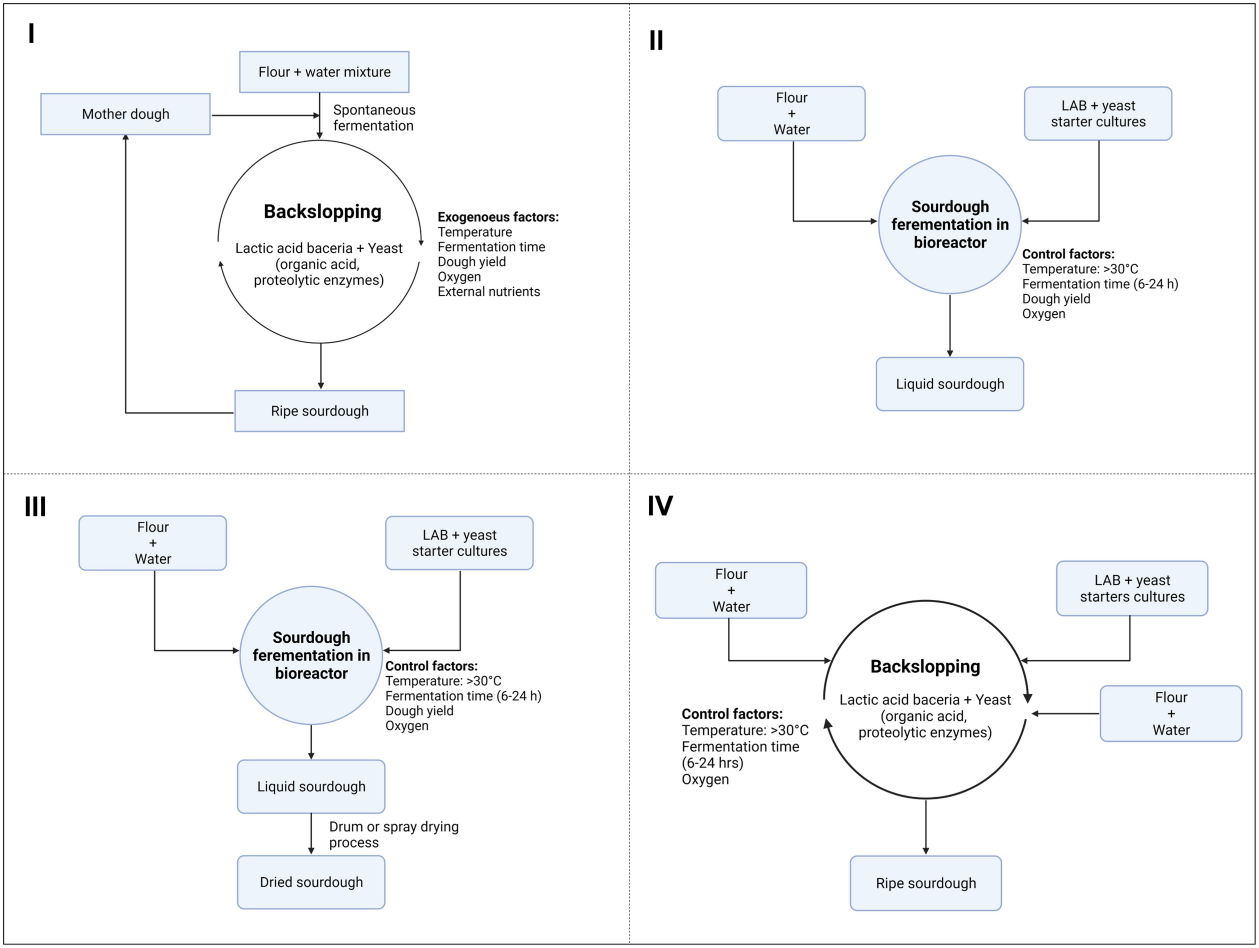
Types of sourdough processes depending on the inocula and the final properties desired for breadmaking.

Nowadays, four types of SD can be made depending on the inocula and the final desired properties of baked goods, such as flavor, texture, smell, stability, and nutritional properties: types I, II, III, and IV (Figure 1; Galli et al., 2019). The type I or traditional SD process depends on the backslopping technique at a low incubation temperature (20°C–24°C), which relies on a repeated cyclic of re-inoculation (6–24 h) with a new batch of flour and water from a previous one derived from a mother dough (Figure 1I). This type of SD is a pure craft, and the dough can be maintained for years. In terms of microbiology, type I SD harbors mixtures of distinctive yeast and LAB species or strains, representing a large diversity of natural SD starters. Backslopping results in the prevalence of the species/strains best adapted to the SD ecosystem. The type of flour used, and its enzymatic, microbiological, nutritional, and textural qualities are of the utmost importance since these factors will determine the stability of the mature doughs (De Vuyst et al., 2014). The main drawback of the type I process is that it cannot be scaled up for industrial exploitation; moreover, it is time-consuming, requires trained and qualified staff, and is not fully controllable (Galli et al., 2019).

In the case of the type II process, doughs are characterized by fermentation with specific LAB strains (Figure 1II). Type II SD is generally not suitable for dough leavening but is used for dough acidification and as a flavor enhancer. This process is shorter than that of type I, with a unique fermentation step of 15–20 h, followed by a storage period of many days. The SD is generally liquid, and fermentation occurs in bioreactors or tanks. Thus, type II SD can be scaled to an industrial level (Corsetti, 2013). In this case, defined acid-tolerant LAB starter cultures are used (e.g., strains of *Levilactobacillus brevis*, *Limosilactobacillus fermentum*, *Lactiplantibacillus plantarum*, *Limosilactobacillus reuteri*, or *Fructilactobacillus sanfranciscensis*), accompanied or not by yeasts, most commonly *Saccharomyces cerevisiae*, which is often added to the final stage but also can be added along with the LAB. Most strains are selected on the basis of their potential to quickly cause acidification of the dough and/or generate specific flavor compounds. These two properties offer a clear added value, which is reflected in the commercialization of dried SD powders used as flavorings in bread production (De Vuyst et al., 2017).

Type III SD is remarkably similar to type II, with the difference being in the dehydration or pasteurization of the liquid-stabilized SD (Figure 1III). Various dehydration techniques can be applied, with drum and spray drying being the most common ones. The starter cultures are selected on the basis of their high acidification capacity, ability to produce flavor compounds, and resistance to the drying procedure. The most common species include heterofermentative *L. brevis* and facultative heterofermentative *Pediococcus pentosaceus* and *L. plantarum* strains (Papadimitriou et al., 2019). Type III SD presents several advantages, such as a long shelf life, a smaller volume, and ease of handling for transportation and storage, which makes it more convenient for industrial bakeries, and finally, the production of standardized end-products. Generally, type III SD is used as an acidifier or as a bread improver (Corsetti, 2013).

Type IV SD is initiated with a LAB starter culture, followed by traditional backslopping as in type I SD (Figure 1IV; Preedy and Watson, 2019). This starter culture-backslopped SD approach is equally characterized by a pattern of three-step succession of LAB communities. However, competition frequently occurs between the added LAB starter culture and the spontaneously growing microorganisms. Differences in stress tolerance may lead the predominance of the autochthonous LAB species and elimination of the LAB starter culture. This may occur because the starter culture is not well adapted to the SD ecosystem and the prevailing conditions (De Vuyst et al., 2017).

Type II and III SD can simplify the production process, but they do not guarantee the same distinctive properties as type I SD; nevertheless, they contain only LAB and require the addition of bakery yeasts as a second step. Recently, liquid SD has been introduced in bakeries as a new technology trend. This type of SD allows the addition of both LAB and yeasts in a single step and thus meets the industrial demand for a more controllable and large-scale SD process (Galli et al., 2019).

## Role of LAB in sourdough fermentation

Sourdough is a very complex ecosystem, where heterofermentative LAB are the dominant organisms and co-exist synergistically with yeasts, which are well adapted to the prevailing acidic environment and can grow to high concentrations [10^7^ colony forming units (CFU)/g], albeit lower than those of LAB (10^8^ CFU/g; De Vuyst et al., 2014). More than 90 different LAB species have already been isolated from SD, including obligately and facultatively heterofermentative species and some homofermentative species, as shown in Table 1.

The fermentation process generates mainly acids, alcohols, aldehydes, esters, and ketones; it is the primary route of volatile compound formation in SD and bread crumb (Limbad et al., 2020). The contribution of LAB to the flavor of SD bread is associated with the production of lactic acid (fresh acidity) and acetic acid (sharp acidity). That also contributes to the amino acid accumulation (e.g., accumulation of glutamate, which is responsible for the umami taste), and the generation of 2-acetyl-1-pyrroline as an end metabolite that is responsible for the aroma of the crust and formed through the Maillard reaction of ornithine in the arginine deiminase (ADI) pathway. Additionally, LAB activity is involved in the accumulation of peptides such as glutathione and glutamyl dipeptides, responsible for the kokumi taste (De Vuyst et al., 2017). The conversion of amino acids such as phenylalanine (sweet), isoleucine (acidic), glycine, serine, and alanine (vinegar/sour) to aldehydes and ketones can form additional flavor compounds (Limbad et al., 2020).

Regarding the metabolic pathways of LAB, the ones that influence bread quality are linked to central carbon flux and limited by cofactor availability, which affects the redox potential of the environment and inside the cell. Homo-and heterofermentative LAB differ fundamentally in the reduced cofactors they regenerate, such as nicotinamide adenine dinucleotide (NAD) or nicotinamide adenine dinucleotide phosphate (NADP). Furthermore, the use of co-substrates, such as oxygen or fructose, as electron acceptors by obligate heterofermentative LAB is coupled with an increase in acetate production in doughs. Thus, the different metabolic requirements of homo-and heterofermentative LAB produce other effects on the redox reactions in SD that influence the quality of the final bread beyond the formation of acetate (Chavan and Chavan, 2011).

The available carbohydrates in wheat flour are maltose, followed by sucrose, glucose, and fructose, along with some trisaccharides such as maltotriose and raffinose. The glucose concentration increases during fermentation because other complex carbohydrates are metabolized by LAB and yeasts; however, yeasts cannot ferment the disaccharide maltose, which is instead fermented by LAB (Chavan and Chavan, 2011). Starting from glucose, homofermentative LAB produce lactic acid through glycolysis, while heterofermentative LAB generate, besides lactic acid, CO_2_, acetic acid, and/or ethanol (De Vuyst et al., 2017). Moreover, carbohydrate metabolism leads to the development of antimicrobials, flavor compounds, and EPS.

On the other hand, the generation of oligopeptides and amino acids is made possible by endogenous flour proteases that become activated at low pH and reduce the gluten disulfide bonds caused by LAB acidification and glutathione reductase activity (De Vuyst et al., 2017). As described earlier, amino acid conversion contributes to the acid stress response, redox balancing, and flavor formation. Protein acid accumulation due to peptide hydrolysis stimulates SD flavor formation and nutrient enrichment (Gobbetti et al., 2014). Phenolic compounds and lipids are only minor compounds in cereal flours. Nevertheless, some LAB species catalyze the release of bound phenolic compounds through feruloyl esterase, tannase (tannin acyl hydrolase), and glycosyl hydrolases, which hydrolyze esters of ferulic acid, galloyl esters of gallotannins, and flavonoid hexoxides, respectively. Phenolic compounds and flavonoids can be further converted into flavor precursors; these conversions are grounded in specific phenolic acid decarboxylases and cinnamic acid reductases of LAB that are associated with cereal flours (De Vuyst et al., 2017). Sorghum and millet flours contain a high level of polyphenols. Their use is suggested for the metabolism of phenolic compounds by microorganisms, specifically by LAB species capable of degrading these compounds, such as *L. brevis, L. helveticus, L. plantarum,* and *P. pentosaceus* (Gänzle, 2014). Regarding fatty acids, fatty acid hydratases can transform oleic acid and linoleic acid into hydroxy fatty acid chains (Gänzle, 2014), whereas phytase hydrolysis, which releases inorganic phosphate and makes minerals bioavailable during the SD process, is primarily dependent on endogenous cereal phytases that become activated by acidification; LAB of the SD microbiota have also been shown to possess phytase activity (De Vuyst et al., 2017).

## Role of yeasts in sourdough fermentation

Yeasts, by producing CO_2_, act as the primary leavening agents in bread products. Baker’s yeasts exhibit a rapid fermentative metabolism and are resistant to many stress factors that are present during breadmaking. The properties of these microorganisms, mainly *S. cerevisiae,* have significant economic and technological value (Chiva et al., 2021). The stability of the SD process depends on cooperation between certain species of LAB and yeasts (Palla et al., 2020). The use of baker’s yeast as a leavening agent has been developed as an alternative for elaborating SD in industrial bread production, where time and speed of production are important factors. Nowadays, baker’s yeast is sold as various product types with improved shelf-life, osmotolerant properties, retention of activity at low temperatures, and flavor generation. The presentation of yeasts includes liquid cream or small granules, compressed as blocks, and dried yeasts that may be dry active or dry frozen (De Vuyst et al., 2017). The yeast variability in SD is affected by dough hydration, type of cereal, and leavening temperature, among others (Chavan and Chavan, 2011; Urien et al., 2019).

The carbohydrates present in flour are fermented *via* glycolysis and further pyruvate breakdown to generate CO_2_ and ethanol. Ethanol also impacts the dough properties, strengthening the gluten network, but a significant proportion evaporates during baking (Rezaei et al., 2015). Both glycerol and succinate are SD osmoprotectants, reducing pH and influencing dough rheology by improving gas retention and gluten formation (De Vuyst et al., 2021). Besides their leavening ability and influence on the strength of the gluten network, yeasts contribute largely to flavor development in bread. This depends not only on one yeast strain but also on symbiosis with the LAB strains present in the matrix (De Vuyst et al., 2017). Yeasts can, for instance, generate flavor metabolites *via* the Ehrlich pathway by converting branched-chain amino acids into higher alcohols and their esters (Pico et al., 2015). Additionally, yeasts produce low levels of organic acids, such as acetic and succinic acids, that contribute to slight acidification of the leavened dough and affect the final flavor (Jayaram et al., 2013). Furthermore, yeasts contribute to antioxidant activity based on the availability of phenols in the flour cereal (Wang et al., 2014). The dephosphorylation of phytate *via* the action of phytases and the antifungal activities of yeast due to the generation of ethyl acetate lead to the release of phenolic compounds (De Vuyst and Neysens, 2005). Moreover, yeasts can also increase the polyphenol content in cereal products by releasing bound and conjugated phenolic acids to free forms after cell wall degradation processes (Palla et al., 2020).

As well, yeasts in SD may present phytase activities which contribute to enhancing the bioavailability of minerals (calcium, iron, zinc, and magnesium), by degrading the phytate-mineral complex that is an antinutritional factor abundant in wholemeal flours (Chiva et al., 2021). Another activity related with the metabolism of yeasts in SD is their ability to increase the content of vitamin B_2_ (riboflavin) in doughs and breads, specifically when *S. cerevisiae* is used as starter culture (Batifoulier et al., 2005). Likewise, baker yeast (*S. cerevisiae*) has been exploited by the potential of synthesize Vitamin D. Concentration of vitamin B_9_ has also been reported to increase during SD fermentation of oat, barley, and rye, particularly by the presence of *S. cerevisiae* and *K. humilis* as starter cultures (Kariluoto et al., 2014). Yeasts also improves the digestibility of bread by the presence of proteases, since during dough fermentation, the proteolysis release small peptides and free amino acids, which are important for rapid microbial growth, acidification, as precursors for the flavor development of leavened baked products, and better protein digestibility (Kaur et al., 2008). Additionally, this proteolytic activity might be used as a tool to reduce certain allergen compounds like gluten, since cereal proteins are one of the most frequent causes of food allergies (Poutanen et al., 2009).

## Presence of postbiotic-like components in sourdough fermentation

In the fermentation process of SD, the microorganisms involved produce a significant quantity of cells and metabolites, which can be in the intracellular or extracellular matrix, and such microorganisms can potentially benefit the consumer, even when present in their non-viable form after baking (Barros et al., 2020). Furthermore, during the baking process, the cell lysis of microorganisms delivers cellular debris that may also have beneficial properties (Sadeghi et al., 2019). Besides whole non-viable microbes, among the examples of postbiotic-like compounds present in SD are SCFAs, secreted proteins/peptides, bacteriocins, secreted biosurfactants, amino acids, flavonoids, EPSs, vitamins, organic acids, and other widely diverse molecules. Other potential postbiotics are residues of the cell debris such as peptidoglycan-derived muropeptides, surface-protruding molecules (pili, fimbriae, flagella), cell-surface associated proteins, cell wall-bound biosurfactants, and cell supernatants (Nataraj et al., 2020). Table 2 presents the main postbiotic components that may occur in foods and the potential health benefits in the host.

**Table 2:** Health benefits and characteristics of the main postbiotic components.

Postbiotic	Main description	Health benefits in the host	Reference
Biosurfactants	Molecules synthesized during the late log or early stationary phase of the growth cycle. Amphiphilic molecules that are composed of glycolipids, lipopeptides, phospholipids, neutral lipids, polysaccharide-protein complexes, and free fatty acids	Disruption and prevention of biofilm formation by pathogenic microorganisms. Wetting, foaming, and emulsification properties, which help the pathogen to adhere, establish itself, and subsequently communicate in biofilms	Nataraj et al., 2020
Exopolysaccharides	Extracellular biopolymers synthesized or secreted by microorganisms during the exponential phase. Based on their monosaccharide composition, exopolysaccharides are further classified into homopolysaccharides and heteropolysaccharides	Biofunctional attributes such as antioxidant, cholesterol-lowering, immunomodulatory, and anti-aging effects; gut microbiota modulation; and anti-toxic, anti-biofilm, and antitumoral effects in preclinical trials	Lynch et al., 2018 and Nataraj et al., 2020
Short-chain fatty acids	Fatty acids with fewer than six carbon atoms in their chains. The most common are acetate, propionate, formate, and butyrate. LAB synthesize SCFAs from non-digestible carbohydrates. Also, bifidobacteria can synthesize short-chain fatty acids, for example acetate and formate	Management of inflammatory bowel disease and colorectal cancer due to their potentiality to overcome the inflammation and proliferation of cancerous cells	Gill et al. (2018) and Nataraj et al. (2020)
Teichoic acids	These are anionic glycopolymers that play key roles in determining the cell shape, regulation of cell division, and other fundamental metabolic aspects of cell physiology. Teichoic acids are generally of two kinds: lipoteichoic acids and wall teichoic acids	Antibiofilm actions against oral and enteric pathogens, immunomodulatory potential, and decreased leaky gut and inflammation	Barros et al., 2020 and Nataraj et al., 2020
Bacteriocins	LAB produce an array of extracellular antimicrobials that inhibit both pathogenic and spoilage-causing microorganisms	Inhibitory potential against various urogenital and antibiotic-resistant pathogens	Bartkiene et al., 2020 and Nataraj et al., 2020
Cell-free supernatant	Cell-free supernatant of LAB is a consortium of low-molecular-weight (hydrogen peroxide, organic acids, carbon dioxide, and di-acetylene) and high-molecular-weight (bacteriocins) compounds	Bioliquid-detergent that reduces the adhesion and biofilm formation of pathogens to various surfaces	de Almada et al., 2016 and Nataraj et al., 2020
Peptidoglycan	Peptidoglycan is a linear glycan strand cross-linked by peptides. The strands are constructed by bonding N-acetylglucosamine and N-acetylmuramic acid	Immunomodulatory, anti-proliferative, and anti-tumor effects	Nataraj et al., 2020
Cell-surface proteins	Proteins that are found in the plasma membrane or in the cell wall. They can be classified into four categories: proteins anchored to the cytoplasmic membrane, lipoproteins, proteins containing a C-terminal motif, and non-covalently bound proteins associated with the cell wall	Immunomodulatory action, secretion of antibacterial peptides, anti-inflammatory effects, anti-adhesion effects, strengthening of epithelial barrier properties, and biosorption of toxic heavy metals	Shigwedha et al., 2014 and Nataraj et al., 2020

The formation of postbiotic-like compounds during SD fermentation has mostly been described for LAB. Many metabolic pathways are activated during fermentation to produce bioactive substances that inhibit pathogenic bacterial growth and prevent bacterial toxin formation. Moreover, LAB produce low-molecular-weight organic components from the metabolism of carbohydrates, amino acids, vitamins, organic acids, and fatty acids. The LAB strain diversity and metabolic capability of generating a variety of bioactive compounds depend on many fermentation parameters, such as pH, time, and temperature. In the case of pH, many differences between SD with a pH of 4.0 and those with a lower pH have been described. When the pH is maintained at 4.0, the presence of lactic acid isomers and ethanol increases the metabolic activity of different LAB and the production of postbiotic-like compounds. By contrast, lower pH values lead to the accumulation of flavor-enhancing volatile compounds. Therefore, optimization of SD pH is a critical determinant of the type of dough obtained, depending on whether the aim is to produce a certain flavor or desired metabolites (Păcularu-Burada et al., 2020b).

Baking is carried out at a temperature up to 200°C–250°C and for an average duration of 1 h (Petrova and Petrov, 2020). Thermal treatment for the generation of postbiotic-like components may be influenced by the type of microorganisms, growth stage, prior exposure to stress, pH value, water activity, and heating mode (conduction, convection, and/or radiation), among other factors. Regarding postbiotics, thermal treatments have been reported to increase cell coarseness and roughness, influencing the immune-modulating properties. The higher the temperature applied, the greater the roughness and degree of coarseness of the cell-surface compared to that of viable cells. Furthermore, adhesion is another property affected by the temperature; when the temperature is higher, the adhesion capacity is reduced. These findings are relevant to the development of processes for postbiotic generation because they indicate that each potential microorganism demands a different temperature and time for inactivation (de Almada et al., 2016).

Several studies have reported the possible postbiotic components in foods (Table 2); however, very few have detailed the functionality and health benefits in the context of SD. Therefore, we will focus on specific compounds such as EPSs, antimicrobial molecules, and fatty acids. The most relevant studies addressing this topic are shown in Table 3.

**Table 3:** Compounds synthetized during sourdough fermentation and in the final bread.

**Compounds**	**Findings**	**Species**	**Source**	**Reference**
Exopolysaccharides	Maximum value of exopolysaccharides synthesized in sourdough with ~22 g/L	*Lactobacillus* spp. *Leuconostoc* spp.	Whole wheat flour Sesame seeds	Păcularu-Burada et al., 2020b
Antibacterial compounds	Inhibitory properties of LAB species against the 15 pathogenic and opportunistic bacterial strains tested through diameter of inhibition zones	*Lactiplantibacillus plantarum* *Lacticaseibacillus casei* *Latilactobacillus curvatus* *Lacticaseibacillus paracasei* *Loigolactobacillus coryniformis*	Rye wheat flour	Bartkiene et al., 2020
Antifungal compounds	Most of the isolated sourdough LAB displayed antifungal activities against seven selected mold strains	*Lactobacillus* spp. *Leuconostoc* spp. *Enterococcus* spp. *Pediococcus* spp.	Rye wheat flour	Bartkiene et al., 2020
Acidification capacity	Strains of *Lactobacillus* spp. showed the highest acidification capacity	*Lactobacillus* spp.	Whole wheat flour	Păcularu-Burada et al., 2020b
Bacteriocins	Five strains were found to produce distinct bacteriocin-like inhibitory substances, but *L. lactis* showed a broader inhibitory range	*Lactiplantibacillus pentosus* *Lactiplantibacillus plantarum*	Rye wheat flour	Gomaa 2013
Fatty acids	*L. hammesii* converts linoleic acid in sourdough, and the resulting monohydroxy octadecenoic acid exerts antifungal activity in bread	*Levilactobacillus hammesii*	Wheat flour	Black et al., 2013
Vitamin B_2_	Strains of *Meyerozyma* spp. overproduce vitamin B_2_ and may increase the nutritional value of the doughs	*Meyerozyma* spp.	Wheat flour	Chiva et al., 2021
Phytase activity	*S. cerevisiae* IMA L15Y and L10Y showed the highest phytase activity when inoculated in the red-grained wheat	*S. cerevisiae* IMA L15Y and L10Y	Red-grained wheat	Palla et al., 2020

Exopolysaccharides are often produced during the SD process. The EPS yield can be correlated to many factors, such as the composition of macronutrients and micronutrients of the substrate, temperature, pH, agitation, and the bacterial strains with defined biochemical properties (Păcularu-Burada et al., 2020a). The EPSs generated by LAB during SD have techno-functional aspects related to their ability to bind water and retain moisture. In the last decade, the use of EPS-producing cultures has attracted the attention of the bakery industry because of the hydrocolloid nature of these polysaccharides, as well as their health benefits, including anticarcinogenic effects (Lynch et al., 2018). Screening of EPS-producing SD strains is limited to wheat and rye fermentations. The SD process offers a convenient means by which the EPS-producing nature of LAB can be exploited to produce baked goods with enhanced quality (Lynch et al., 2018). This is typically achieved by adding a pre-fermented SD starter with defined EPS-producing strains, commonly on a 10%–40% (w/w) flour basis, to the final bread dough mix (Galle and Arendt, 2014).

Exopolysaccharide from SD provides an opportunity to improve consumers’ health. These compounds stimulate carbohydrate fermentation to SCFAs by the intestinal microbiota. For example, dextran is metabolized by gut microbes to acetate, butyrate, and propionate. Propionate has been postulated to have several beneficial effects, such as reducing cholesterol and triglyceride levels, and increasing insulin sensitivity. Furthermore, oligosaccharides can act as soluble receptor analogs of epithelial cell-surface carbohydrates and inhibit pathogens or bacterial toxin adhesion to epithelial surfaces, an initial stage of an infective process. Thus, the large variety of oligosaccharides produced by LAB enzymes, also involved in EPS production, makes LAB potential candidates for preventing infection or inflammatory bowel diseases (Galle and Arendt, 2014). Several factors, such as dough yield, fermentation time, pH, sucrose content, and the fermentation substrate, influence the amount of EPS formed *in situ*. It was reported that glucan formation by *L. reuteri* from sucrose was higher in softer doughs, probably because of better diffusion of the substrates and extracellular glycansucrases (Seitter et al., 2019). Exopolysaccharides yield was also improved when sucrose was added stepwise (fed-batch) to the fermenting dough. Furthermore, with the pH adjusted to a constant value of 4.7, the EPS level increased. Interestingly, fermentation with wheat flour, a rye–wheat mixture, or rye bran with 10% sucrose addition showed that EPS production was the most efficient and fastest when rye bran was supplied as a substrate (Di Monaco et al., 2015).

Some examples of LAB metabolites with antimicrobial activity are bacteriocins, organic acids, bacteriocin-like inhibitory substances, and others. Bacteriocins are well-known peptides/proteins synthesized by bacterial ribosomes, and their function is to either kill or inhibit the growth of closely related bacteria. These antimicrobial molecules produced by LAB are usually regulated by environmental conditions and the productive strain growth phase. Bacteriocin production is initiated in the early log phase, suggesting that bacteriocin is a primary metabolite. In the case of other antimicrobial compounds from LAB, it is very important to emphasize that these metabolites are released after 48 h of fermentation (Păcularu-Burada et al., 2020b). The inhibitory effect on pathogenic or spoilage microorganisms varies widely. The effects of some antimicrobial metabolites produced by two LAB (*L. plantarum* and *Lactobacillus delbrueckii*) cultivated on wheat dough extracts were studied. The species *L. plantarum* had a stronger inhibitory effect against *Penicillium* spp., whereas weaker inhibition was observed against *Aspergillus niger*. These LAB species were also used to produce SD bread, where good resistance against fungal contamination was observed. Another study with isolated LAB strains from spontaneous rye SD (belonging to species such as *Leuconostoc mesenteroides, Latilactobacillus curvatus,* and *Levilactobacillus brevis*) showed no inhibitory effect against *Aspergillus* spp. and weak inhibition of *Penicillium* spp. However, it was found that all strains showed satisfactory inhibition of *Bacillus cereus* (Păcularu-Burada et al., 2020b).

In the case of fatty acids, many LAB can produce SCFA, especially acetate, propionate, and butyrate, with potential therapeutic effects on depression, autism, anxiety, and stress (Gill et al., 2018; Păcularu-Burada et al., 2020a). Additionally, metabolites from the conversion of fatty acids by specific LAB strains may contribute to the prolonged storage life of sourdough bread. Black et al. (2013) demonstrated that *Levilactobacillus hammesii* converts linoleic acid to a monohydroxy octadecenoic acid, preventing fungal spoilage of bread without adversely impacting on the sensory properties. This conversion was observed in SD fermentation supplemented with linoleic acid as a substrate (Black et al., 2013). These conversions from fatty acids have not been fully explored in SD. It remains unknown whether hydroxy fatty acids produced by LAB could have a specific positive impact on health.

## Effect of heat treatment on microbial viability

The application of probiotics in bread is unlikely because the high temperature and dehydration that occur during the baking process terminate cell viability. Besides, to ensure the benefits of probiotics, the number of LAB must exceed 10^6^ CFU/ml or per gram at the time of consumption (Zhang et al., 2016, 2018). The factors that affect the behavior of probiotics during baking include the temperature, moisture content, and structure of the matrix. Many studies have been performed to determine the effect of thermal and dehydration kinetics on the inactivation of the microorganisms in SD bread during baking. Furthermore, most research mainly studied the impact of the baking conditions, storage, and composition on the technological quality of the baked bread instead of the microbiological quality (Duc Thang et al., 2019).

The kinetics of bread baking is an essential factor in the formation of the crust and the crumb, which give bread its textural and sensory properties. The minimum time required for baking bread is when 98% of the starch in the dough is completely gelatinized, and this time decreases as the baking temperature increases. For example, the baking time for a dough of 60 g at 175°C is 9 min. Under the same conditions, but at a temperature of 200°C, the minimum baking time is reduced to 6 min. However, when the temperature increases to 235°C, the baking time decreases by only 1 min. The viability of microorganisms is affected by the minimum baking time because of the heat and dehydration stresses to which they are exposed. If the baking time is shortened by increasing the baking temperature or reducing the bread size, higher residual viability of LAB species may be obtained after baking (Debonne et al., 2018).

Zhang et al. (2018) evaluated the viability of *L. plantarum* at three different baking temperatures (174°C, 200°C, and 235°C). The results showed that the viable *L. plantarum* counts were reduced by up to 4–5 log CFU/g, while the initial viable count in the dough was 8.8 log CFU/g (Zhang et al., 2018). The kinetics of the baking process showed that in the first 2 min, the microorganisms were only slightly inactivated, with a reduction of <0.5 log CFU/g; between minutes two and six, the reduction in the bacteria count was exponential, with a decrease of 3 log CFU/g; and in the last 2 min, the kinetic changed into a stationary phase with a maximum decrease of 1 log CFU/g (Zhang et al., 2016, 2018). In the case of yeasts, Debonne et al. (2018) found that the yeast count decreased from an initial value of 9 log CFU/g to 4 log CFU/g with baking at 200°C for 13 min. Under different conditions, 150°C for 8 min, the yeast count was reduced by only 2–3 log CFU/g, impacting the product’s shelf life. Debonne et al. (2018) observed that the use of SD resulted in negligible detection of spore-forming bacteria, with 3 log CFU/g being the highest recorded value, after heat treatment (150°C, 8 min).

*In vitro* metabolic studies on the effect of thermal treatment on the viability of microorganisms and the functionality of their components already exist, but they are scarce. Nachtigall et al. (2021) examined the impact of thermal treatment on the molecular mass of EPS synthetized by *Streptococcus thermophilus*. The EPS solution was analyzed at 60°C, 80°C, and 90°C for 10, 30, and 60 min each and cooled to room temperature in an ice bath. When the EPS was untreated, the average molecular mass was 2.90 × 10^6^ Da. In the case of thermal treatment at 60°C and 80°C for up to 60 min, the molecular mass of the EPS was not significantly affected. Similar behavior occurred at 90°C and 10 min residence time, with no reduction of the molecular mass. However, after 30 min of residence time, the molecular mass decreased significantly to 2.40 × 10^6^ Da, and after 60 min, to 2.40 × 10^6^ Da (Nachtigall et al., 2021). Therefore, a prolongated thermal treatment affects the molecular composition of EPS. Unfortunately, there is scarce information about EPS stability at baking temperatures (160°C–220°C). More studies are necessary to determine if the functionality of EPS in bread is compromised by the thermal treatment.

The functionality of antimicrobial compounds has also been analyzed by exposure to high temperatures. Păcularu-Burada et al. (2020b) evaluated the functionality of antibacterial and antifungal compounds treated at 60°C, 80°C, and 121°C for 15 min, forming inhibitory ratios or halo zones. The antimicrobial-compound-producing LAB were grown in three different types of flour extract: buckwheat, chickpea, and quinoa. *Bacillus* spp. was used as the pathogen for antibacterial activity, and *A. niger, Aspergillus flavus,* and *Penicillium* spp. were the microorganisms assessed for antifungal activity. The stability test showed that the antifungal capacity after the thermal treatments differed depending on the type of flour used. In the case of chickpea flour, neither strain inhibited the three fungal species. For buckwheat treated at 80°C, various heat-stable compounds with an inhibitory effect against *Penicillium* spp. were present. The fermented quinoa flour extract showed the highest inhibition ratio of 17% after thermal treatment at 60°C (15 min). However, at 121°C, an inhibitory effect against *A. niger* was observed with an inhibition ratio of 7.20% (Păcularu-Burada et al., 2020a). This result is in line with those obtained by Varsha et al. (2014), who concluded that antifungal compounds produced during fermentation are resistant to sterilization temperatures and have a high value in the baking industry (Varsha et al., 2014). Regarding antibacterial activity, the flour extracts fermented with both strains showed thermally stable antibacterial compounds that inhibited *Bacillus* spp. (Varsha et al., 2014). Similar results were found by Cizeikiene et al. (2013), who concluded that many antibacterial compounds, especially bacteriocins, are resistant to thermal treatment and may have many functions in foods that are exposed to high temperatures, such as baked goods (Cizeikiene et al., 2013). However, experimentation with higher temperatures, up to 220°C, is needed to evaluate the stability of the antifungal and antibacterial activity.

In the case of other compounds (e.g., biosurfactants, cell-surface proteins, cell-free supernatants, SCFAs, etc.), there are not sufficient studies to describe their properties and stability after thermal treatment. Therefore, further research is necessary to understand the functionality of these compounds after a baking process using *in vitro* and *in vivo* models.

## Effects of sourdough bread on health

Different studies evaluated the potential health benefits of sourdough bread (SDB) consumption in healthy volunteers and in subjects with metabolic or gastrointestinal diseases. Table 4 summarizes some studies of SDB with different formulations and doses that evaluated the impact of their ingestion in healthy volunteers and patients with pathologies such as irritable bowel syndrome (IBS), among others. The administration of different SDB formulations resulted in reduced glycemic responses compared with a traditional white wheat bread leavened with *S. cerevisiae*, where the content of blood glucose after 120 min of digestion was 125 mmol/L for white bread and 90 mmol/L for SDB (Scazzina et al., 2009; Bondia-Pons et al., 2011). Scazzina et al. (2009) evaluated the influence of SD on starch digestibility in bread. Four experimental formulations made from two wheat flours by two leavening techniques (SD and with *S. cerevisiae*) were analyzed. Sourdough bread showed higher resistant starch levels and significantly lower glycemic responses in young, healthy subjects. The reduction in the glycemic response was not related to the starch hydrolysis rate; however, organic acids produced by the SD microbiota might delay gastric emptying without affecting starch accessibility (Scazzina et al., 2009). Likewise, in Bondia-Pons et al. (2011), SD fermented rye bread was tested in healthy subjects. Sourdough bread contained a higher level of total fiber and free phenolic acids, a higher starch hydrolysis rate, and a lower postprandial insulin response. Sourdough bread altered plasma amino acids and their metabolites. The increase in tryptophan precursors following consumption of SD rye bread might stimulate a higher tryptophan concentration, resulting in lower appetite and food intake. Likewise, the levels of picolinic acid, which catabolizes the tryptophan metabolism associated with pro-inflammatory functions, significantly decreased after SD rye bread intake (Bondia-Pons et al., 2011).

**Table 4:** Evaluation of the benefits of sourdough bread in human health and disease.

**Product**	**Dose**	**Model**	**Findings**	**Reference**
Two SDB (different fermentation times: 4 h and 24 h) compared with yeast-fermented bread	One slice of bread (80 g) at 3-week intervals	**Clinical trial** (36 healthy volunteers aged 20–31 years) Double-blind	Highest satiety with SDB fermented for 24 h. In both SDB: faster gastric emptying, lower glycemic responses, higher concentration of total free amino acids and better digestibility	Rizzello et al., 2019
SDB (>12 h fermentation) compared with yeast-fermented bread	Six slices of the study bread (150 g)/day for 7 days	**Clinical trial** (26 patients with IBS aged 18–65 years) Double-blind	Higher reduction in ATIs to their monomeric form Lower levels of FODMAPs	Laatikainen et al., 2017
Whole-grain rye SDB (40 h of fermentation) compared with yeast-fermented crispbread and unfermented rye crispbread	One slice (59.4 g)	**Clinical trial** (24 healthy adults aged 18–70 years) Single-blind, cross-over trial	Higher satiety and degradation of *β*-glucans	Zamaratskaia et al., 2017
SDB with low FODMAPs against regular rye SDB	3.5–4 slices (105–120 g) of each bread/day in the 1st week 7–8 slices of SDB (210–240 g) per day from the 2nd to the 4th week	**Clinical trial** (87 patients with IBS aged 18–65 years) Double-blind, placebo-controlled cross-over study	Control of IBS symptoms and reduction of gastrointestinal gas accumulation Increase in dietary fiber intake and good acceptance	Laatikainen et al., 2016
Whole-grain rye SDB compared with WB and rye-bran-enriched WB	6–10 slices (25–30 g/slice) In two 4-week test periods	**Clinical trial** (21 healthy subjects with mild gastrointestinal symptoms aged 38–65 years); Cross-over study	Lower postprandial insulin concentration Improvement in the first-phase of insulin secretion Increase in postprandial concentrations of SCFAs	Lappi et al., 2014
Wholegrain wheat SDB against WB	6 slices (for women) and 7 (for men) of SDB per day (162.5 g) for 6 weeks	**Clinical trial** (14 normoglycemic/normoinsulinemic adults and 14 hyperglycemic/hyperinsulinemic adults aged 43–70 years); Crossover study	Improvement in glucose iAUC in response to an OGTT within hyperglycemic/hyperinsulinemic subjects	MacKay et al., 2012
Endosperm rye SDB compared with standard WB	One portion (50 g) at intervals of 1–2 weeks	***In vitro*** Starch and protein hydrolysis **Clinical trial** (16 healthy subjects aged 23 ± 3.7 years)	Higher levels of total fiber and phenolic acids and a higher starch hydrolysis rate Lower postprandial insulin response Beneficial changes in plasma amino acids and their metabolites	Bondia-Pons et al., 2011
Wholemeal wheat SDB (19.5 h fermentation) compared with WB, wholemeal wheat, and wholemeal wheat + xylanase	One slice with crust (50 g) of the test breads	**Clinical trial** (11 insulin-resistant subjects, aged 40–65 years)	Lowest postprandial glucose and insulin responses	Lappi et al., 2010
Two SDB compared with yeast-fermented wholemeal bread and yeast fermented WB	One slice (50 g)	***In vitro*** Starch hydrolysis **Clinical trial** (8 healthy volunteers aged 23–25 years)	Significantly lower glycemic responses in SDB Resistant starch levels were higher in the SDB	Scazzina et al., 2009
Whole wheat SDB (3 h fermentation) compared with whole wheat barley bread and WB	One slice (50 g)	**Clinical trial** (10 overweight male subjects) Single-blind, cross-over	Lower overall glucose and GLP-1 responses Lower glucose iAUC	Najjar et al., 2008
SDB (24 h fermentation) with 4 flours compared with yeast-fermented 4-flour bread	A portion of 80 g for 2 days	**Clinical trial** (17 celiac sprue patients) Double-blind	Intestinal permeability not significantly different from baseline in 13 of the 17 patients	Di Cagno et al. (2004)

SDB, Sourdough bread; WB, white bread; IBS, irritable bowel syndrome; ATIs, alpha-amylase/trypsin inhibitors; FODMAPs, Fermentable, Oligo-, Di-, and Mono-saccharides and Polyols; SCFAs, short-chain fatty Acids; OGTT, oral glucose tolerance test; iAUC, incremental area under the curve; GLP-1, glucagon-like peptide 1.

Moreover, SDB elicited a weaker insulin response in overweight human males, as well as insulin-resistant, and hyperglycemic/hyperinsulinemic patients (Najjar et al., 2008; Lappi et al., 2010; MacKay et al., 2012). Najjar et al. (2008) analyzed the effect of SD-fermented whole wheat bread in overweight male patients. Sourdough bread was associated with the least disturbance in carbohydrate homeostasis, as well as lower overall glucose and glucagon-like peptide-1 (GLP-1) responses, indicating that its consumption could promote the prevention and management of metabolic disorders associated with type 2 diabetes (Najjar et al., 2008). Lappi et al. (2010) evaluated the postprandial insulin response to five different bread formulations in insulin-resistant patients. Sourdough bread (19.5 h of fermentation) showed the lowest postprandial glucose and insulin responses. The proteolysis in SD may be the contributory factor (Lappi et al., 2010). Finally, MacKay et al. (2012) reported that SDB showed an improvement in incremental area under the glucose curve in response to an oral glucose tolerance test (OGTT) in patients with hyperglycemia/hyperinsulinemia, which represents a potential to positively influence postprandial glucose responses in people at risk of cardiovascular disease (MacKay et al., 2012).

Moreover, SDB can increase the postprandial concentrations of SCFAs (Lappi et al., 2014). Sourdough has been shown to change the nutritional quality and health effects of grain ingredients (Lappi et al., 2014). Lappi et al. (2014) compared the impact of three bread formulations (SD rye bread, white bread with rye bran, and white bread) on glucose metabolism and SCFA plasma levels in healthy subjects with mild gastrointestinal symptoms. Sourdough rye bread lowered the postprandial insulin concentration and improved first-phase insulin secretion. The improvement in first-phase insulin secretion and the reduction of hyperinsulinemia in the later postprandial phase may prevent alterations in glucose metabolism. The consumption of both rye bran formulations improved subjects’ gastrointestinal quality of life. Additionally, SD rye bran bread increased postprandial concentrations of butyrate and propionate (Lappi et al., 2014).

Sourdough bread is also considered to have beneficial effects on postprandial satiety (Zamaratskaia et al., 2017; Rizzello et al., 2019). Rizzello et al. (2019) and Zamaratskaia et al. (2017) tested the impact of SDB in healthy subjects and obtained the highest satiety response; however, only Rizzello et al. (2019) showed a lower glycemic response. Wholegrain SD rye bread led to the highest satiety (Zamaratskaia et al., 2017). Wholegrain rye bread is linked to a reduced molecular weight of arabinoxylans and *β*-glucans. Fibers with high solubility and low molecular weight might increase their fermentability and satiety. Additionally, organic acids produced in SD could affect satiety (Zamaratskaia et al., 2017). Rizzello et al. (2019) evaluated two SDBs with different fermentation times (4 and 24 h) in healthy subjects. Results showed that SDB fermented for 24 h obtained the highest fullness perception in the shortest time. Furthermore, both SDBs exhibited faster gastric emptying, a lower glycemic response, a higher total free amino acid concentration, and better digestibility than yeast-fermented bread (Rizzello et al., 2019).

Additionally, SDB has the potential benefit of improving some enteropathies such as celiac sprue (CS) or irritable bowel syndrome (IBS; Di Cagno et al., 2004; Laatikainen et al., 2016, 2017). Di Cagno et al. (2004) evaluated SDB formulations for tolerance by CS patients, while Laatikainen et al. (2016, 2017) focused on specific characteristics of SD that would benefit IBS patients. Di Cagno et al. (2004) included selected SD lactobacilli for their ability to hydrolyze albumin, globulin, and gliadin fractions during SD fermentation in SDB formulated with four flours (wheat, oat, millet, and buckwheat flour). The result was improved tolerance of SDB with 30% wheat flour and no significant alterations in the intestinal permeability values in 13 of the 17 patients (Di Cagno et al., 2004). In the case of IBS patients, Fermentable, Oligo, Di-, and Mono- saccharides, and Polyols (FODMAPs) are considered triggers of IBS symptoms. Low-FODMAP SDB was administered and evaluated against regular SDB. Low-FODMAPs SDB improved IBS symptoms and reduced gastrointestinal gas accumulation, presenting lower fermentation in the colon, flatulence, abdominal pain, and stomach rumbling; it also increased dietary fiber intake and was well accepted by patients (Laatikainen et al., 2016). Likewise, SDB presented a greater reduction in alpha-amylase/trypsin inhibitors (ATIs) to their monomeric form and showed lower levels of FODMAPs than yeast-fermented bread; ATIs and other non-gluten proteins are associated with a pro-inflammatory effect on intestinal epithelial cells, which could cause gastrointestinal symptoms (Laatikainen et al., 2017). Sourdough bread applications have been demonstrated to improve health and even to aid in the management of symptoms of sensitive pathologies such as CS and IBS. However, further studies are needed to characterize and elucidate the mechanisms of action of these benefits and to perform additional clinical trials considering other diseases and a more diverse demographic.

## Future perspectives

Recent studies are attempting to characterize the different compounds and cell debris present in SD. Among the examples are SCFAs, EPSs, biosurfactants, bacteriocins, cell-free supernatants, and cell-surface proteins, and the main health benefits are anti-immunomodulatory, antioxidant, and antimicrobial effects.

The potential health benefits promote consumer preference for foods prepared with LAB. This increasing need for the formulation of functional food products containing LAB starter cultures from sources such as kefir, yogurt, or kombucha, which have reported health benefits, has led to the application of different consortiums as mixed starters for SD breadmaking (Limbad et al., 2020; Plessas et al., 2020). Starters have a key role in the physical characteristics of SDB, modifying raw bread dough and cooked bread products (extensibility, elasticity, viscosity, and the organoleptic properties of the final bread; Fujimoto et al., 2019; Reese et al., 2020; Landis et al., 2021). There are numerous microbial consortiua with potential health benefits that need to be assessed. Another research opportunity is related to ingredients. Organic farming and products are gaining increasing consumer demand due to their association with sustainable production. Recent studies tested the impact of flour selection by evaluating new organic flour options created to meet consumers’ needs (Urien et al., 2019; Pontonio et al., 2021). The use of organic flours may offer better functional characteristics, such as a higher total free amino acid content, than non-organic flour (Pontonio et al., 2021). Likewise, researchers are analyzing the effect of ingredients and the environment on the SD microbiota. Factors such as the bakers’ skin microbiota may have an important role in the composition of bacteria and fungi in starters (Reese et al., 2020). Also, cereal fermentation may potentially improve nutritional quality and health effects; there is a wide variety of cereals and pseudocereals that can be combined and evaluated to obtain different profiles for fiber content and potential postbiotic-like components (Koistinen et al., 2018; Păcularu-Burada et al., 2020a). However, a noticeable challenge is to reach a consensus for applying standardized culture-based techniques to characterize SD microbial diversity to minimize the variability and biases among studies and define criteria for evaluation of SD starters and their bread products.

## Conclusion

Postbiotic-like components in SD provide potential beneficial health properties such as better digestibility, satiety, antioxidant properties, among others. More studies are needed with a defined microbiological consortium and ingredients, to generate a SD able to apport specific nutrients and health benefits; therefore, it is imperative that the different postbiotic forms present in the final dough be characterized, their yield measured, and their potential health properties probed by *in vitro* and *in vivo* studies.

## Author contributions

TG-C: conceptualization and final supervision. OP-A and AZ-H: investigation and writing and original draft preparation. OP-A and TG-C: visualization. LG-A, TR, GV, and TG-C: review and editing. All authors contributed to the article and approved the submitted version.

## Funding

The authors acknowledge the financial support by Tecnologico de Monterrey within the funding program “Fund for financing the publication of Scientific Articles.” This work was also supported by i-Link+2019 program from the Spanish National Research Council (CSIC) and Tecnologico de Monterrey (Project: LINKB20023).

## Conflict of interest

The authors declare that the research was conducted in the absence of any commercial or financial relationships that could be construed as a potential conflict of interest.

The reviewer MT declared a shared parent affiliation with the author TR to the handling editor at the time of review.

## Publisher’s note

All claims expressed in this article are solely those of the authors and do not necessarily represent those of their affiliated organizations, or those of the publisher, the editors and the reviewers. Any product that may be evaluated in this article, or claim that may be made by its manufacturer, is not guaranteed or endorsed by the publisher.
